# Assessing a New Method for Measuring Fetal Exposure to Mercury: Newborn Bloodspots

**DOI:** 10.3390/ijerph13070692

**Published:** 2016-07-09

**Authors:** Jessica W. Nelson, Betsy L. Edhlund, Jean Johnson, Christina E. Rosebush, Zachary S. Holmquist, Shanna H. Swan, Ruby H. N. Nguyen

**Affiliations:** 1 Environmental Tracking and Biomonitoring Program, Minnesota Department of Health, St. Paul, MN 55164, USA; jessica.nelson@state.mn.us (J.W.N.); jean.johnson@state.mn.us (J.J.); christina.rosebush@state.mn.us (C.E.R.); 2 Public Health Laboratory, Minnesota Department of Health, St. Paul, MN 55164, USA; betsy.edhlund@state.mn.us; 3 Division of Epidemiology & Community Health, School of Public Health, University of Minnesota, Minneapolis, MN 55454, USA; zsholmquist@gmail.com; 4 Department of Preventive Medicine, Icahn School of Medicine at Mount Sinai, New York, NY 10029, USA; shanna.swan@mssm.edu

**Keywords:** mercury, biomonitoring, newborn bloodspots, cord blood, fetal exposure

## Abstract

Background: Measuring mercury in newborn bloodspots to determine fetal exposures is a novel methodology with many advantages. Questions remain, however, about its reliability as an estimate of newborn exposure to mercury. Methods: We studied mercury concentrations in paired bloodspots and cord blood from a convenience sample of 48 Minnesota women and infants. Results: The limit of detection for bloodspots was higher than for cord blood (0.7 and 0.3 μg/L in bloodspots and cord blood, respectively) with the result that mercury was detected in only 38% of newborn bloodspots compared to 62% of cord blood samples. The geometric mean mercury concentration in cord blood was 0.6 μg/L. Mercury concentrations were almost uniformly lower in bloodspots than in cord blood (mean ratio (±SD) = 0.85 ± 0.4), their mean value was significantly less than that for the cord blood (*p* = 0.02), and the two methods were highly correlated (*r* = 0.82). Conclusion: These preliminary findings indicate that newborn bloodspot mercury measurements have utility; however, until bloodspot analyses are more sensitive, they are likely to underestimate *in utero* exposure.

## 1. Introduction

Fetal exposure to mercury is a public health concern because even small amounts of mercury can damage the developing brain and nervous system [1]. Mercury exposure may affect future learning abilities, memory, and attention, and lead to learning and behavioral problems later in life [1]. Due to these adverse health effects, it is in the interest of local and state health departments to create methods of monitoring neonatal exposure to mercury for the purposes of identifying populations at risk, determining sources of exposure, and evaluating the need for targeted public health interventions.

A novel approach to assessing the magnitude of fetal exposure to environmental chemicals is the analysis of newborn bloodspots, which uses small amounts of blood collected from an infant’s heel on a filter card soon after birth. Bloodspots are routinely collected for state newborn screening programs, which test them for treatable health conditions not evident at birth [2,3]. Compared to the standard measures of prenatal exposure that use cord blood or maternal blood, biomonitoring using bloodspots offers advantages of ease of collection and storage, as well as decreased cost. Due to these advantages, interest in using newborn bloodspots for biomonitoring is growing for a variety of exposures; recent studies have measured mercury and other metals, cotinine, and perfluorochemicals in this specimen type [4,5,6,7,8,9].

Laboratory methods for measuring mercury concentrations in bloodspots are relatively new. Only a few state health agencies have reported measuring mercury in newborn bloodspots [4,5,8,9]. A previous study from the Minnesota Department of Health (MDH) measured mercury in bloodspots from newborn infants born to mothers living around the Lake Superior Basin and found that 10% of the 1126 Minnesota infants tested had mercury concentrations greater than the level corresponding to the U.S. Environmental Protection Agency’s (EPA) reference dose (RfD) for methylmercury of 5.8 μg/L [5]. A population-based state-wide investigation of mercury in newborn bloodspots in Utah found that 1% of the 5915 bloodspots tested had mercury levels above 5.8 μg/L [8].

While numerous studies have examined the relationship between mercury found in cord blood and maternal blood using paired samples [10], the association between mercury concentrations in newborn bloodspots and those in cord blood is unknown. Before public health surveillance and clinic-based studies can be conducted using newborn bloodspot methodology, the reliability of newborn bloodspots as a method to measure mercury exposure in newborns must be assessed. The newborn bloodspot–cord blood association is particularly important as the RfD for methylmercury is based on cord blood measurements [1]. We aimed to determine the association between total mercury in paired newborn bloodspots and cord blood in a convenience sample of women and infants from the Minneapolis/St. Paul metropolitan area. In doing so, our purpose was two-fold: to investigate the utility of using newborn bloodspot biomonitoring to conduct public health surveillance of population exposures to mercury, and to explore a feasible methodology for state health departments to identify the occurrence of high mercury exposures.

## 2. Materials and Methods

The human subjects’ approval was granted by the Institutional Review Boards of the University of Minnesota-Fairview and the MDH; participants provided written consent for themselves as well as their newborns.

Study participants were sampled from an ongoing National Institutes of Health (NIH)-funded prospective pregnancy cohort, The Infant Development and Environment Study (TIDES). TIDES participants were pregnant women recruited between 2010 and 2012, with infants born no later than the end of January 2013. The aim of TIDES was to determine the association of an environmental toxicant during pregnancy with the genitourinary anatomy of neonates, and to provide normative data on these anatomic measures. Details of TIDES can be found elsewhere [11]. For the current study, women who were enrolled in TIDES between June and December 2012 and who had not yet delivered were eligible. Seventy-two women were approached during their scheduled third trimester visit for TIDES and informed about the new study. Sixty-one (85%) eligible and interested women provided separate consent at this visit, and we received at least one biological sample from a total of 55 (76%) mother-infant pairs. Demographic and dietary (fish consumption) information collected in surveys during a third trimester prenatal visit were included in the analysis.

Using a protocol developed as part of the National Children’s Study [12], the clinician attending the woman’s birth collected the cord blood sample soon after the delivery of the placenta. Briefly, a needle was used to collect cord blood from the umbilical vein into a blood tube pre-screened for mercury. Blood collected in the first tube was discarded to avoid metal contamination from the collection needle. Clotted specimens and those of low volume (<1.5 mL) were deemed not acceptable a priori; however, no samples were rejected due to either of these reasons.

Newborn bloodspot samples were collected specifically for the purpose of this study from infants 24–48 h after birth, immediately following the routine collection of bloodspots for the MDH Newborn Screening Program. The same laboratory technician collected bloodspots for the MDH program and this study using standard procedures [13]. Two spots were collected for each infant on bloodspot filter cards (Whatman Neonatal Cards, GE Healthcare Life Sciences, Pittsburgh, PA, USA, and were air dried on a clean counter in the hospital per newborn screening procedure for 24 h and then placed in a plastic envelope. All cord blood and newborn bloodspot specimens were stored in a −20 °C freezer until laboratory analysis, which occurred between four and seven months after collection.

Laboratory analysis of mercury from bloodspots and cord blood was performed at the MDH Public Health Laboratory. Using a method described previously, researchers took two 3 mm punches from the bloodspot card—ensuring that the area of the filter card containing ink was not punched—and mercury was extracted from the punches during overnight digestion. The resulting solution was analyzed using inductively coupled plasma mass spectrometry (ICP-MS) [5]. Most punches were taken from near the edge of the bloodspot; previous method validation found no variation in mercury according to the location of the punch. Mercury concentrations were calculated using a blood volume of 3.1 µL blood per punch, for a total of 6.2 µL blood per specimen [14].

In addition, immediately following each specimen collection, we used punches from the blank area of the card as a control for each sample. There were no samples with detectable levels in the blank punches. One sample duplicate per batch of 20 samples was analyzed. Additional quality control measures included routinely spiking a National Institute of Standards and Technology (NIST) Standard Reference Material (SRM) onto the filter paper for analysis with each batch. We also analyzed a sample of the same SRM not spotted onto the paper to assess any mercury that may have been lost on the paper. Our recoveries for five sample tests of the SRM are presented in Table 1. We observed similar recoveries regardless of whether the SRM was spotted or unspotted, though the recovery was slightly lower with spotting. Also presented in Table 1 are our results after spotting cards with spiked blood; however, these spiked concentrations were not certified. For the four different mercury levels of blood spotted onto cards, we saw acceptable recoveries across the calibration range (e.g., none less than 77%).

Cord blood samples were analyzed for mercury via ICP-MS after dilution [15]. The method detection limit (MDL) was 0.7 μg/L for mercury in bloodspots compared to 0.3 μg/L for mercury in cord blood. Hematocrit was measured in cord blood by the University of Minnesota-Fairview Laboratory. All samples were analyzed using the PerkinElmer ELAN DRC II.

To determine whether differences observed between mercury levels in bloodspot and cord blood samples could be related to the differing laboratory methods used, an analysis of split cord blood samples was performed. For subjects with mercury detected in both the original cord blood and the corresponding bloodspot measurement, the cord blood samples were spotted onto filter paper, dried on a bench top in a cleanroom, and then analyzed for mercury.

The split sample experiment that spotted cord blood onto filter paper and analyzed these bloodspots for mercury was limited to 15 samples. This analysis was further complicated by the fact that mercury concentrations in these samples were low, with many near the MDL of 0.7 µg/L. All 15 cord blood samples in the experiment had mercury concentrations above the MDL, but when these samples were spotted onto filter paper, only four had concentrations above the MDL (results not shown). The bloodspot samples had low recoveries when compared to the cord blood measurements.

For statistical analysis, mercury concentrations were log-transformed to produce normality. For non-detect values, we substituted the MDL divided by the square root of 2. The Pearson correlation coefficient for the bloodspot and cord blood samples was determined using log-transformed values; the same log-transformed values were used in the paired *t*-test to determine whether the mean bloodspot differed significantly from the mean cord blood level. All analyses were performed using SAS v. 9.2 (SAS, Cary, NC, USA).

## 3. Results

The average age of the women in this study was 32 years; the participants were fairly homogenous in that they were highly educated, had relatively high incomes (56% had annual household incomes greater than $75,000), and were predominantly white (92%) (results not shown).

Forty-eight matched pairs of newborn bloodspot and cord blood samples were available. Mercury was detected in 18 (38%) bloodspot and 30 (62%) cord blood samples (Table 2). Sixteen pairs had detectable mercury in both specimens. The geometric mean (GM) mercury concentration in cord blood was 0.6 μg/L; no GM for mercury in bloodspots was calculated due to the low detection frequency. The 95th percentile mercury concentrations were 2.6 μg/L in bloodspots and 3.4 μg/L in cord blood. Only one participant had a mercury concentration above 5.8 μg/L, measured in both types of specimens.

Mothers who reported eating two seafood meals per week during their third trimester had higher cord blood mercury concentrations than those who reported eating no seafood meals (GM 1.1 vs. 0.3 μg/L, respectively, *p* < 0.01). Mothers who had a graduate degree or completed graduate work had slightly higher cord blood mercury concentrations than those with fewer years of education (GM 0.8 vs. 0.4 μg/L, respectively, *p* < 0.05).

**Figure 1:**
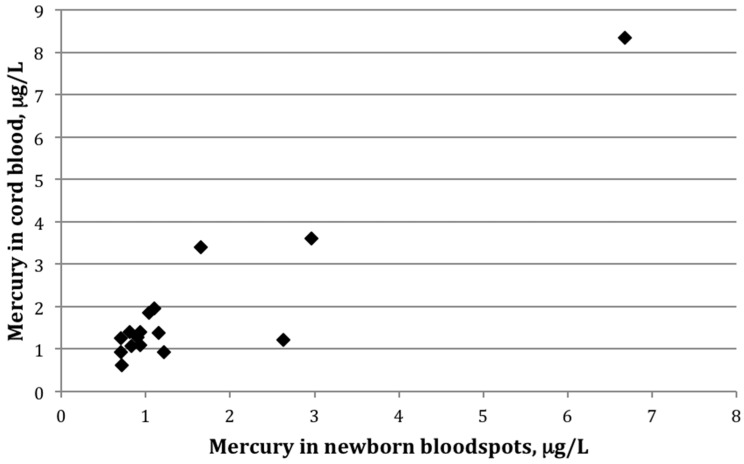
Scatterplot showing mercury (μg/L) in cord blood versus newborn bloodspots among 16 mother-infant pairs in which mercury was detected in both cord blood and newborn bloodspot (*r* = 0.82, *p* < 0.01).

**Figure 2:**
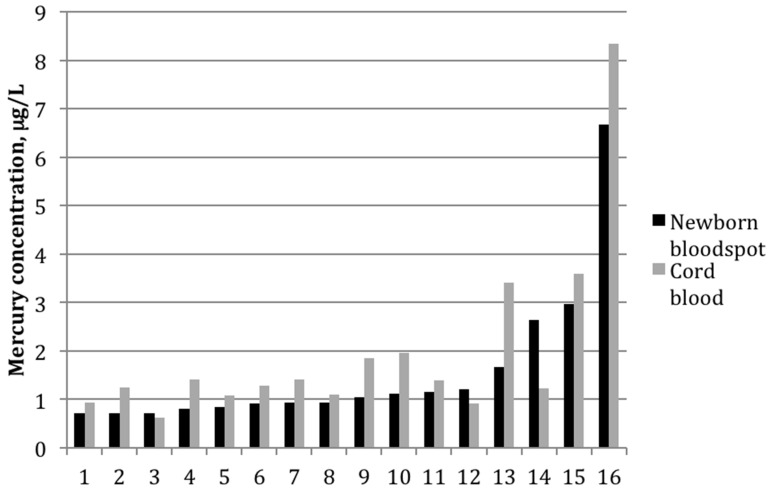
Mercury concentrations (μg/L) for each mother-infant pair in which mercury was detected in both cord blood and newborn bloodspot (*n* = 16).

Among the 16 paired samples with detectable mercury in both bloodspot and cord blood, the average bloodspot-to–cord blood ratio was 0.85 ± 0.4 (range: 0.5–2.2). The paired *t*-test indicated that the mean values between the two groups differed significantly (*p*-value = 0.02). The two sample types were also strongly correlated (Figure 1), with a Pearson correlation coefficient on log-transformed values of 0.82 (*p* < 0.01). Figure 2 shows mercury concentrations for each bloodspot–cord blood pair within this group of 16 participants. In all but three sample pairs, the cord blood was higher than what was determined from the bloodspot; for one sample, the bloodspot level was more than double that of the cord blood value.

## 4. Discussion

While mercury concentrations in paired newborn bloodspot and cord blood samples were strongly correlated in this study, they were significantly lower, approximately 15% on average, in bloodspots than in the corresponding cord blood. This finding suggests that analysis of newborn bloodspot specimens for mercury may systematically underestimate fetal exposure. As the EPA reference level for methylmercury is based on measurements in cord blood [1], this value may not be an appropriate health-based reference level to use in relation to mercury concentrations reported in newborn bloodspots. However, there was some contradictory evidence in three of our samples, in which the bloodspot value was either similar or—in the case of one sample—drastically more than that of the cord blood. Although we performed additional quality control analysis on this outlying sample, we found no explanation for this finding.

The results of our split experiment on cord blood samples spotted onto filter paper, though limited by small sample size and low mercury concentrations, indicated that the differences in the laboratory method may contribute to the differences observed between cord blood and newborn bloodspot mercury concentrations. The mercury in cord blood samples was extracted with a simple extraction diluent, whereas the mercury in the bloodspot samples was extracted from blood that was dried on filter paper; this may lead to lower recovery levels compared to the cord blood analysis. Although the recoveries of a standard reference material (SRM) for the two sample types were comparable and acceptable, the SRM contains a relatively high mercury concentration; similar experiments have not yet been performed on an SRM at a mercury concentration closer to the concentrations found in this study. While we ran initial experiments with blood spiked onto cards at lower mercury concentrations that had similar recoveries, these tests were not certified and mercury concentrations were still higher than those seen in the great majority of study bloodspot and cord blood samples. Before conclusions can be drawn, however, more research is needed in studies with larger sample sizes.

Our findings could also reflect a biological difference, though this possibility is more difficult to explain given that cord and spot blood were collected within 24–48 h of each other and the half-life of methylmercury in blood is ~50 days [1]. We explored whether hematocrit could be a biological factor involved in the relationship, as the higher mercury concentrations in cord blood compared to maternal blood have been attributed to higher hematocrit levels in the developing fetus than in the mother [10]. Our exploratory analysis (results not shown) did not find evidence that hematocrit modified the association between bloodspot and cord blood mercury concentrations, though our findings were limited by the small sample size. Hematocrit measured in cord blood was not correlated with cord mercury or the bloodspot-to–cord blood mercury ratio. In regression models that examined cord blood versus bloodspot mercury concentrations, adjusting for hematocrit did not alter the *R*^2^ value, nor was hematocrit a significant predictor in these models.

Our preliminary data indicate that these urban Minnesota infants had cord blood mercury concentrations similar to those in several other populations studied in North America [16,17]. With some caution due to our limited sample, we found lower concentrations in our small Minnesota population than in other locations including New York City (New York), Honolulu (Hawaii), and Baltimore (Maryland) [18,19,20,21]. The latter studies included substantial proportions of infants born to non-white mothers and found that certain racial groups, such as African Americans and some Asian populations, had higher exposures than whites. In Rhode Island, infants born to African American mothers had GM mercury concentrations of 2.1 μg/L, compared to 0.5 μg/L for the total sample [16]; for infants born to China-born Asian women in New York City, the GM concentration was 12.6 μg/L, compared to 4.4 μg/L in the total sample [19]. By contrast, the GM concentration in our small Minnesota population was 0.6 μg/L. Our study included women who were predominantly white and had relatively high incomes, with fewer women from the racial/ethnic groups that, based on other studies, appear to be at increased risk for mercury exposure, potentially due to higher consumption of mercury-containing fish [18,19,20].

The purposes of our study were to investigate population mercury exposures using bloodspot biomonitoring to conduct public health surveillance, and to explore a feasible methodology for state health departments to identify high exposures. To do so we used the most common procedures for collecting newborn dried blood samples, which were collected 24–48 h after the collection of the cord blood. This time discrepancy, although realistic, limits our reliability measures. In addition, our results are limited by the study’s small sample size. In particular, due to the high frequency of non-detection in our newborn bloodspot samples, the number of bloodspot–cord blood pairs with detectable mercury concentrations in both sample types—the sub-population in which our main analysis was performed—was small (*n* = 16). This limits the generalizability of our prevalence estimates and makes it difficult to assess the performance of the bloodspot method in identifying high mercury exposures with regard to sensitivity, specificity, and positive predictive value. This methodology should be replicated in future studies in larger populations and in those with higher mercury exposures to adequately compare population samples and identify the prevalence of high mercury exposure using newborn bloodspots.

Our future studies will aim to more thoroughly understand the discrepancy between the lower mercury concentrations in newborn bloodspot samples compared to paired cord blood samples—as well as the circumstances behind the opposite occurrence in a small proportion of samples—using a larger and more diverse population, and to explore the variability we observed in the bloodspot-to–cord blood ratio. While the average ratio was 0.85, individual results varied four-fold. With a larger sample size, future work will also better assess the performance of bloodspots as a method to identify high mercury exposures. In the bigger picture, MDH will continue to monitor mercury and other environmental exposures in Minnesotans, with a focus on pregnant women, children, and certain racial/ethnic groups who may be most vulnerable to these exposures.

In conclusion, these preliminary findings provide evidence that, although newborn bloodspots can be used to estimate in utero exposure to mercury, there may be a systematic underestimation of mercury exposure using our methodology. Our study emphasizes the need for more research on the capacity of newborn bloodspots as a reliable and non-invasive tool to measure heavy metals, both in research settings as well as in public health biomonitoring programs. Ultimately this type of novel technology may improve our ability to conduct and interpret public health surveillance and environmental health research.

## 5. Conclusions

These preliminary findings indicate that newborn bloodspot mercury measurements have utility; however, until bloodspot analyses are more sensitive, they are likely to underestimate *in utero* exposure.

## Figures and Tables

**Table 1:** Performance of dried bloodspot quality control materials.

Sample	Sample Description	True Value	% Recovery
SRM * 966	NIST SRM 966	31.4 µg/L	80%
SRM 966 on cards	NIST SRM 966 spotted onto cards	31.4 µg/L	74%
RLV **	Spiked blood on cards	2.41 µg/L	124%
Patient 1	Spiked blood on cards	15.9 µg/L	88%
Patient 2	Spiked blood on cards	5.36 µg/L	77%

* SRM—Standard Reference Material; ** RLV—Report Level Verification.

**Table 2:** Distribution of total mercury (μg/L) in newborn bloodspots and cord blood from 48 mother-child pairs in Minneapolis, Minnesota, 2012.

Measure	Mercury (μg/L) in Newborn Bloodspots *n* = 48	Mercury (μg/L) in Cord Blood *n* = 48
% non-detect	62%	38%
Method detection limit	0.7	0.3
Geometric mean	N/A *	0.6
Median	ND	0.6
95th percentile	2.6	3.4
Minimum	ND	ND
Maximum	6.7	8.3
Percent > 5.8 μg/L	2%	2%

* N/A because detection frequency was <50%; ND = Non-detect.
